# Clinical effectiveness and cost-effectiveness of the rehabilitation enablement in chronic heart failure facilitated self-care rehabilitation intervention for people with heart failure with preserved ejection fraction and their caregivers: rationale and protocol for a multicentre randomised controlled trial – REACH-HFpEF trial

**DOI:** 10.1136/bmjopen-2024-094254

**Published:** 2025-05-27

**Authors:** Rod S Taylor, Emma Burrell, Claire O’Hare, Elizabeth A Thomson, Anna Placzek, Jessica C Bollen, John G F Cleland, Aynsley Cowie, Hasnain M Dalal, Christi Deaton, Patrick J Doherty, Katie Dudman, Heather Fraser, Julia Frost, Colin Greaves, Nick Hartshorne-Evans, Melvyn Hillsdon, Tracy Ibbotson, Mohammad Jarallah, Kate Jolly, Alex McConnachie, Emma McIntosh, Valerie Smith, Iain Squire, Louise Taylor, Samantha van Beurden, Chim C Lang, on behalf of the REACH-HFpEF Investigators

**Affiliations:** 1 School of Health & Wellbeing, University of Glasgow, Glasgow, UK; 2 Medical School, University of Exeter, Exeter, UK; 3 School of Cardiovascular & Metabolic Health, University of Glasgow, Glasgow, UK; 4 Cardiac Rehabilitation, NHS Ayrshire and Arran, Kilmarnock, UK; 5 Research, Development and Innovation, Royal Cornwall Hospitals NHS Trust, Truro, Cornwall, UK; 6 Primary Care, University of Exeter Medical School, Truro, UK; 7 Cambridge Institute of Public Health, School of Clinical Medicine, University of Cambridge, Cambridge, UK; 8 Health Science, University of York, York, Yorkshire, UK; 9 Health Economics and Health Technology Assessment (HEHTA), University of Glasgow, Glasgow, UK; 10 School of Sport, Exercise and Rehabilitation Science, University of Birmingham, Birmingham, UK; 11 Pumping Marvellous Foundation, Preston, UK; 12 University of Exeter, Exeter, UK; 13 Institute of Health and Wellbeing, University of Glasgow, Glasgow, UK; 14 University of Birmingham, Birmingham, UK; 15 Department of Physical Therapy and Health Rehabilitation, Majmaah University, Al Majma’ah, Saudi Arabia; 16 Institute of Applied Health Research, University of Birmingham, Birmingham, UK; 17 Robertson Centre for Biostatistics, University of Glasgow, Glasgow, UK; 18 University College Dublin School of Nursing Midwifery and Health Systems, Dublin, Ireland; 19 Glenfield Hospital, Leicester, UK; 20 Heart Manual Department, Lothian Health Board, Edinburgh, UK; 21 Division of Cardiovascular Research, University of Dundee, Dundee, UK; 22 University Kebangsaan Malaysia, Bangi, Selangor, Malaysia

**Keywords:** Self Care, REHABILITATION MEDICINE, Heart failure

## Abstract

**Introduction:**

Heart failure with preserved ejection fraction (HFpEF) is common and causes functional limitation, poor health-related quality of life (HRQoL) and impairs prognosis. Exercise-based cardiac rehabilitation is a promising intervention for HFpEF, but there is currently insufficient evidence to support its routine use. This trial will assess the clinical and cost-effectiveness of a 12-week health professional-facilitated, home-based rehabilitation intervention (REACH-HF), in people with HFpEF, for participants and their caregivers.

**Methods and analysis:**

REACH-HFpEF is a parallel two group multicentre randomised controlled trial with 1:1 individual allocation to the REACH-HF intervention plus usual care (intervention group) or usual care alone (control group) with a target sample size of 372 participants with HFpEF and their caregivers recruited from secondary care centres in United Kingdom. Outcome assessment and statistical analysis will be performed blinded; outcomes will be assessed at baseline and 4-month and 12-month follow-up. The primary outcome measure will be patients’ disease-specific HRQoL, measured using the Minnesota Living with Heart Failure questionnaire, at 12 months. Secondary outcomes include patient's exercise capacity, psychological well-being, level of physical activity, generic HRQoL, self-management, frailty, blood biomarkers, mortality, hospitalisations, and serious adverse events, and caregiver's HRQoL and burden. A process evaluation and substudy will assess the fidelity of intervention delivery and adherence to the home-based exercise regime and explore potential mediators and moderators of changes in HRQoL with the intervention. Qualitative studies will describe facilitators’ experiences of delivery of the intervention. A cost-effectiveness analysis (CEA) of the REACH-HF intervention in participants with HFpEF will estimate incremental cost per quality-adjusted life year at 12 months. The CEA will be conducted from a UK NHS and Personal Social Services perspective and a wider societal perspective. The adequacy of trial recruitment in an initial 6-month internal pilot period will also be checked.

**Ethics and dissemination:**

The study is approved by the West of Scotland Research Ethics Committee (ref 21/WS/0085). Results will be disseminated via peer-reviewed journal publication and conference presentations to researchers, service users and policymakers.

**Trial registration number:**

ISRCTN47894539.

**STRENGTHS AND LIMITATIONS OF THIS STUDY**

- The study compares an established rehabilitation programme with usual care for individuals with heart failure and preserved left ventricular ejection fraction and their caregivers.
- Evaluation of a home-based model of intervention delivery that can improve access to rehabilitation services.
- Due to the nature of the intervention, blinding of trial participants and clinicians to group allocation was not possible. Outcome assessment and data analysis were blinded.

## Introduction

 Heart failure (HF) is common, often leads to impaired physical function and reduced health-related quality of life (HRQoL) and increases morbidity, mortality and healthcare costs.^1^,^5^ At least half of people with HF have preserved ejection fraction (HFpEF).^3 6^ In contrast to HF with reduced ejection (HFrEF), for which there are several guideline-recommended pharmacological and non-pharmacological therapies that improve life expectancy and HRQoL, there are few for HFpEF, including sodium-glucose co-transporter 2 inhibitors.^7^ A recent meta-analysis of seven randomised controlled trials (RCTs) involving 346 participants with HFpEF shows that participation in exercise training may improve exercise capacity and HRQoL.^8^ Given the finite nature of this evidence base, larger multicentre trials with longer-term follow-up are still needed to confirm these potential benefits of exercise-based rehabilitation for HFpEF.

The Rehabilitation EnAblement in CHronic HF (REACH-HF) intervention is a comprehensive exercise-based rehabilitation and self-management programme informed by evidence, theory and service user perspectives designed for people with HF and their caregivers.^9^ As a home-based intervention, REACH-HF offers an alternative to traditional centre-based programmes and can improve access and uptake of rehabilitation.^10^ A multicentre RCT showed the REACH-HF programme was clinically effective and cost-effective for people with HFrEF.^11 12^

Additionally, a single centre pilot RCT in 50 participants with HFpEF allocated to receive REACH-HF or usual care alone demonstrated favourable trends, including improvements in disease-specific HRQoL (between group difference in Minnesota Living with Heart (MLwHF) Questionnaire total score (−11.5, 95% CI: −22.8 to 0.3 at 6 months follow-up) and cost-effectiveness.^13^ The pilot study supported the feasibility and acceptability of the REACH-HF intervention for participants with HFpEF and the RCT design.

Accordingly, the REACH-HFpEF trial was designed to investigate the clinical effectiveness and cost-effectiveness of a home, exercise-based rehabilitation programme for patients with HFpEF.

### Aims and objectives

We aim to assess the clinical and cost-effectiveness of REACH-HF plus usual care (intervention) versus usual care alone (control) in participants with HFpEF and their caregivers.

The primary objective is to compare disease-specific HRQoL at 12-month follow-up between participants with HFpEF in the intervention and control groups.

Secondary objectives:

- To check the adequacy of trial recruitment in an initial 6-month internal pilot study.
- To compare the following secondary outcomes between participants with HFpEF in the intervention and control groups at 4-month and 12-month follow-up: exercise capacity, psychological well-being, level of physical activity, generic HRQoL, disease-specific HRQoL, self-management activities, frailty, prognostic biomarker, clinical events (death and hospital admission) and serious adverse events.
- To estimate the cost-effectiveness of REACH-HF, compared with usual care alone, in participants with HFpEF as incremental cost per quality-adjusted life year (QALY) at 12 months post-randomisation.
- To explore the moderators and mediators of change in the primary outcome of participants with HFpEF in the intervention group.
- To qualitatively explore REACH-HF facilitators’ experiences of the delivery of the intervention.
- To compare psychological well-being, HRQoL, self-care activities and burden between caregivers in the intervention and control groups at 4-month and 12-month follow-up.
- To assess the fidelity of delivery of the REACH-HF intervention (to inform further future refinement/implementation in the UK NHS if the intervention is effective).

## Methods and analysis

This protocol is reported in accordance with the Standard Protocol Items: Recommendations for Interventional Trials 2013 guidance.^14^

### Design

**Figure 1:**
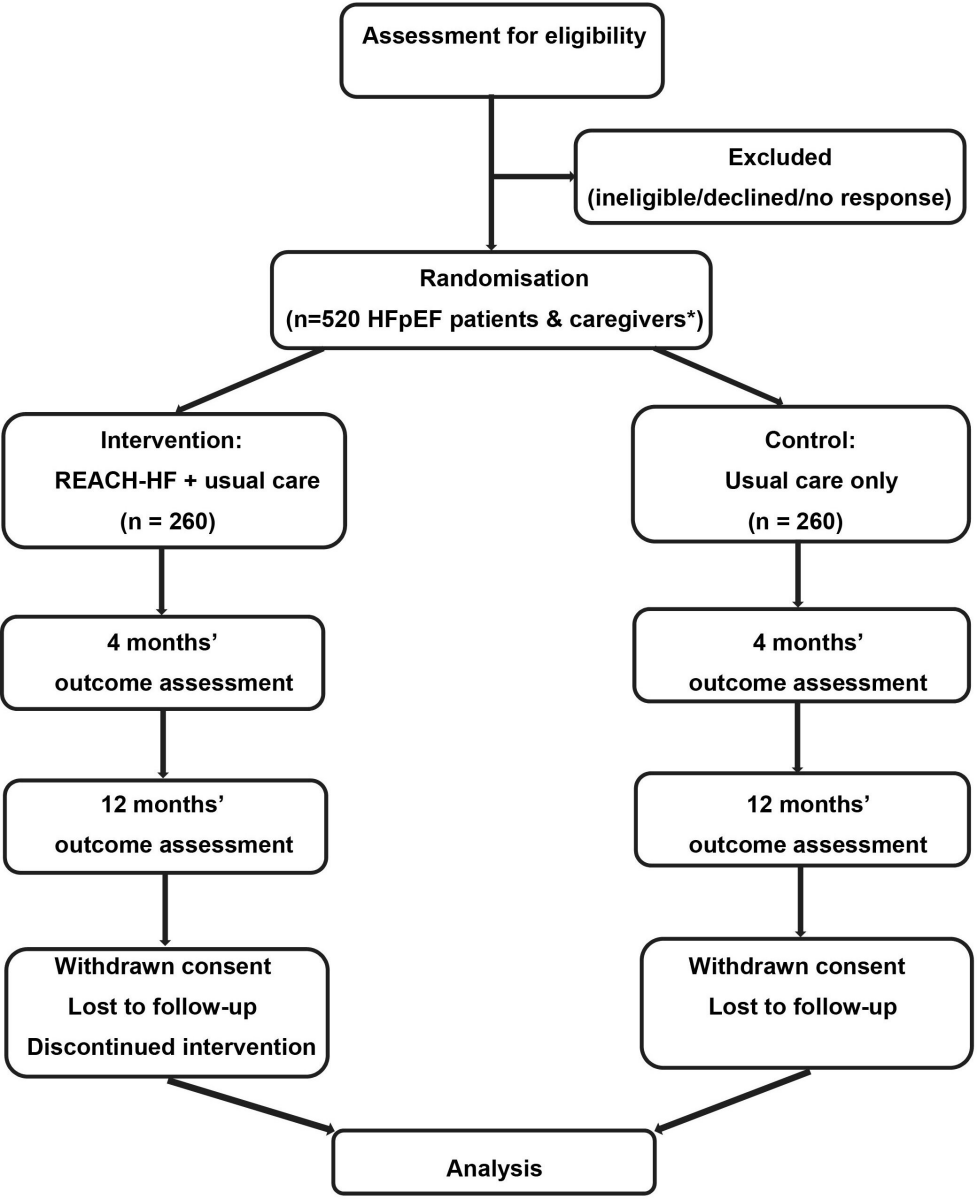
Illustration of study flow. *Dependent on number of caregivers recruited.

REACH-HFpEF is a multicentre parallel two group superiority RCT with nested process and health economic evaluations and an internal pilot phase. Given the complex nature of the REACH-HF intervention, it is not possible to blind participants or those involved in the provision of care beyond the point of randomisation. Researchers collecting outcome data and the statistician undertaking the data analysis will be blinded to treatment allocation to minimise potential bias. The RCT was registered on 15 December 2021 (ISRCTN47894539). An illustration of the study design is shown in figure 1.

### Setting and recruitment

The study plans to recruit a total of 20 sites across England, Northern Ireland, Scotland and Wales. Patients are being recruited from both primary and secondary care pathways including HF registers and outpatient clinics. Follow-up procedures will usually be conducted on NHS premises. Conduct of the study will be led by a local principal investigator, supported by a research nurse or fellow and/or research assistant at each site, all of whom are trained in Good Clinical Practice (GCP) and in the requirements of the study protocol.

We have experienced a slower rate of trial recruitment of 0.8 patient/site/month compared with our predicted rate of 1.5 patients/site/month. As a result, we have implemented a number of strategies: (1) negotiated with our trial funder (NIHR) a 9-month extension to our recruitment closure date; (2) regular communication with our sites including quarterly trials newsletter, a weekly email to all sites of recruitment figures, and termly principal investigator/trial site team web meetings to discuss progress; and (3) introduction of a financial incentive to sites based on successful patient recruitment.

### Study population

**Figure 2:**
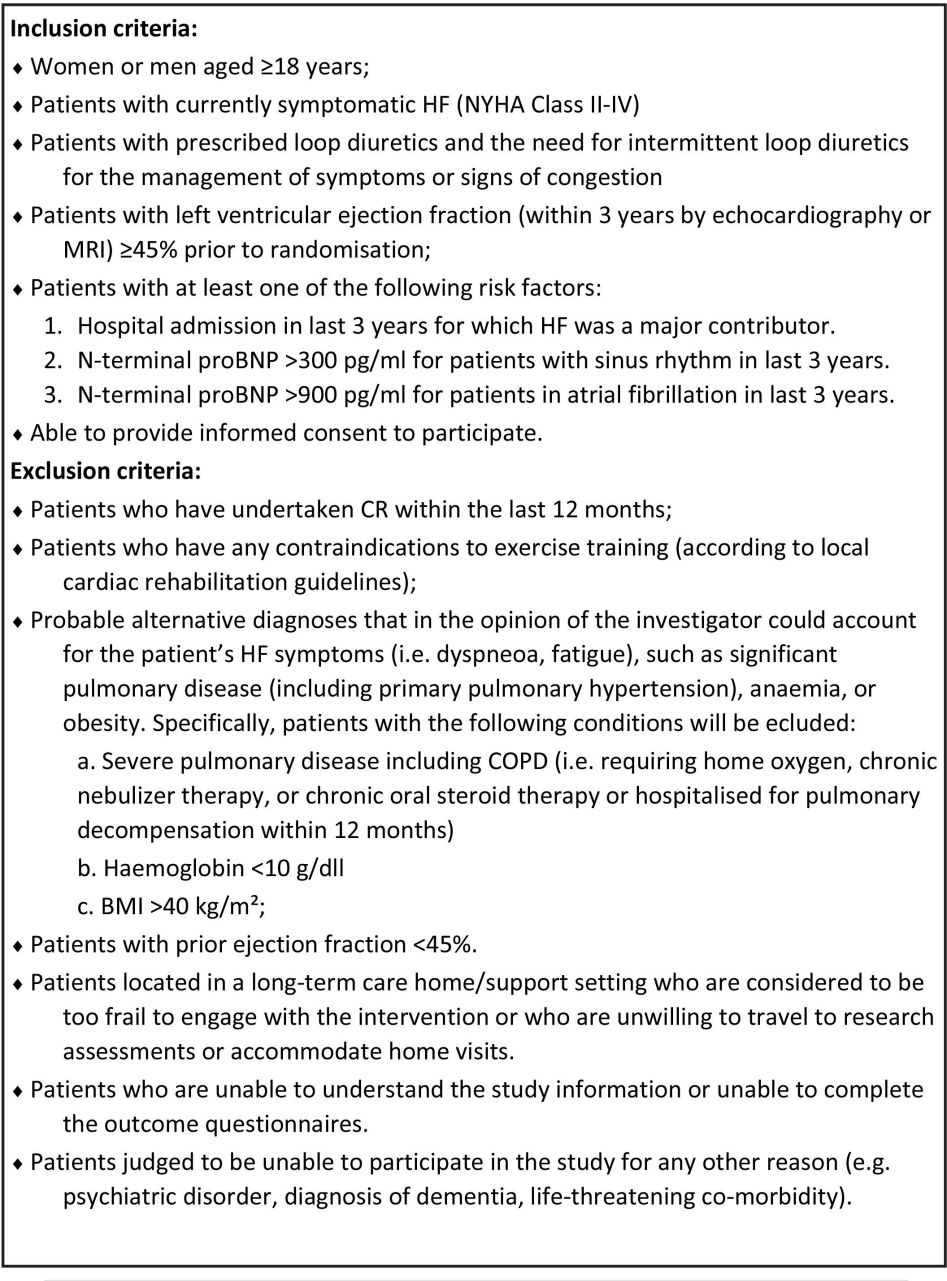
Patient with HFpEF inclusion and exclusion criteria. BMP, body mass index; BNP, B-type natriuretic peptide; COPD, chronic obstructive pulmonary disease; CR, cardiac rehabilitation; HF, heart failure; NYHA, New York Heart Association.

The study population includes eligible patients and caregivers. Participating patients will be aged 18 years or older and have a confirmed diagnosis of symptomatic HF with left ventricular ejection fraction ≥45% within the last 3 years prior to randomisation, confirmed by echocardiography or MRI. Patients who have undertaken cardiac rehabilitation within the last 12 months and those who have any contraindications to exercise training will be excluded. Inclusion and exclusion criteria are detailed in figure 2.

Participants may choose to withdraw at any time and are given the option to fully withdraw from the study, or they can withdraw from the intervention and/or site visits but continue to complete the patient-reported outcome questionnaires only, especially the primary outcome of the MLWHF questionnaire. Data will be collected up to the point of withdrawal and used for analysis. If a participant deviates from the intervention protocol, they will be followed up as intention to treat. Participating caregivers will be aged 18 years or older and provide unpaid support to patients. Participant and carer consent forms are available to view as onlinesupplemental files 1  2.

### Randomisation

Participants will be randomly allocated in a 1:1 ratio to either intervention or control group. Randomisation will be stratified by investigator site and minimised on investigator site, sex and left ventricular ejection fraction (45–55% vs >55%). Randomisation will be achieved by using a secure web-based system. The research team will enter the participant identifier and the system will verify eligibility using data contained in the eCRF (electronic case record form).

### Intervention

REACH-HF is a home-based CR programme providing self-care support to the patient and their caregiver.^9 11 12^ It was developed in cooperation with people living with HF and their caregivers, as well as service providers using an established rigorous intervention development framework^9^ to incorporate existing evidence, clinical guidance on HF self-care, behaviour change theory and key stakeholder perspectives. Table 1 provides an intervention description according to the Template for Intervention Description and Replication checklist.^15^

Details of the exercise component of the intervention are provided in table 2.

**Table 1:** Summary of the REACH-HF intervention description according to the Template for Intervention Description and Replication^15^

Brief name	Rehabilitation EnAblement in CHronic Heart Failure (REACH-HF)
Why	The rationale for REACH-HF was to provide a home-based rehabilitation comprehensive self-care support programme to people with heart failure and their caregivers to help them manage their condition (https://sites.exeter.ac.uk/reachhf/).It was co-created with people living with heart failure (HF) and their families, as well as service providers using an established rigorous intervention development framework to incorporate existing evidence, clinical guidance on HF self-care, behaviour change theory and key stakeholder perspectives (patients, caregivers, service providers and experts in the field).^14^REACH-HF draws on several theoretical perspectives, but key principles included building understanding of the condition to provide a rationale for change (Leventhal’s common-sense model^47^) such as how physical fitness affects heart failure symptoms; building intrinsic motivation and promoting autonomy (self-determination theory^48^); promoting adaptation to living with heart failure and adopting an active rather than passive approach to coping^49 50^; and encouraging learning from experience through engagement in self-regulation activities (control theory^51^). The elements aimed at managing stress and anxiety used psychological intervention processes based on cognitive–behavioural therapy^52^ and mindfulness therapy^53 54^.
What – materials	The REACH-HF intervention includes four core elements: REACH-HF manual for patients with a choice of two structured exercise programmes: a chair-based exercise and a progressive walking training programme (available as a CD and from REACH-HF website) and relaxation programme (available as a CD and from REACH-HF website). Patients are advised to exercise ≥3 times per week, starting from their own personal level and gradually building up over 2–3 months in time/distance/walking pace. Patient ‘Progress Tracker’ – an interactive booklet designed to facilitate learning from experience to record symptoms, physical activity and other actions related to self-care. Patient’s record: (1) how long/far they plan to walk, (2) whether they have done it, (3) how it felt to identify whether they should be moving up or down in efforts next time and (4) their weekly steps per minute (pace). ‘Family and Friends Resource’ – a manual for use by caregivers aimed to increase their understanding of HF and caregiver physical and mental well-being. Facilitation by healthcare staff (eg, nurse, physiotherapist and exercise specialist) experienced in cardiac rehabilitation/heart failure management. The REACH-HF programme was originally designed for patients with HFrEF. However, sections of the manual (including the medication section) have been revised to make it relevant to patients with HFpEF, and an additional section on the nature of causes and treatment of HFpEF has been added.
What – procedures	Patients and caregivers work through the self-help manual over a 12-week period with facilitation involving contact by a specially trained intervention facilitator who will help to assess patient needs and concerns, build the patient’s and caregiver’s understanding of how best to manage HFpEF and provide individually tailored support based on each patient’s identified needs and concerns.
Who provided	REACH-HFpEF trial funding is provided for two/three healthcare professionals with experience of cardiac rehabilitation/heart failure: cardiac rehabilitation nurse, physiotherapist or exercise specialist or HF specialist nurse) from each site, who are responsible for delivering the REACH-HF intervention, and will attend a 2-day web-based training course on the use of person-centred counselling and how to tailor the intervention for the patient and their caregiver, led by clinicians in the Heart Manual Department, NHS Lothian (https://services.nhslothian.scot/theheartmanual/reachhf/).Topics covered in training include: self-management in HF; psychological aspects of HF; health behaviour change; supporting family and caregivers; physical activity and chair-based exercise.
How	The programme has been designed to be delivered over 12 weeks, with a recommended two face-to-face contacts with a REACH-HF facilitator taking place in the patient home and 2–3 follow-up telephone contacts in between.‘Real world’ programme implementation, especially during the COVID-19 pandemic, has resulted in alternative modes of delivery. These have included: combined centre-based and home-based delivery (eg, baseline and end-of-treatment assessments conducted in clinics, with home visits and/or phone support in between) and an entirely remote delivery model, where all sessions (including assessments) were conducted by telephone.
Where	Patient home and/or clinic.
When and how much	Initial face-to-face session: 60–90 min—initial clinical consultation, facilitator discusses programme and introduces patient/caregiver to the REACH-HF resources.Telephone consultations: 2–3 (dependent on patient needs) of~10 mins—check on progress with HF manual and exercise programme.Final face-to-face session: 60–90 min—final clinical consultation, review of goals and plan for continuing REACH-HF programme independently
Tailoring	While the principles of the REACH-HF intervention are the same across HF patients, facilitators are trained to tailor intervention delivery to individual patient needs, for example, adjust exercise level to current fitness.

HFpEF, heart failure with preserved ejection fraction; HFrEF, heart failure with reduced ejection.

**Table 2:** REACH-HF intervention—exercise prescription for chair and walking programme

	Chair based exercise programme (CBE)	Walking Programme (WP)
Duration (support by facilitators)	10–12 weeks	10–12 weeks
Frequencydays/week	2–3 days/week	Progress to 3–4 days/week
Session durationminutes/session	Range 13–40 minLevel 1~13 min includes warm up (WU) and cool down (CD) only *Level 2~21 min (6 min WU and CD)Level 3~21 min (6 min WU and CD)Level 4~25 min (6 min WU and CD)Level 5~28 min (7 min WU and CD)Level 6~30 min (7 min WU and CD)Level 7~38 min (7 min WU and CD)	Progress to 20–30 min (with additional 3–5 min warm up/cool down)Level 1: 5–10 minLevel 2: 10–15 minLevel 3: ≥20 min
Intensity	‘Moderate’The initial exercise training intensity is in the range of 40%–70% of a patient’s capacity. This is ideally based on incremental shuttle walk test (ISWT) or 6-minute walk test (6MWT) calculated metabolic equivalents (METs) prior to commencing the core exercise training component.Each of the seven CBE levels has a known MET value which aligns with roughly 70% of the mean MET score derived from the ISWT and 6MWT. The CBE programme has built-in (on screen) pacing and quality assurance of movement (video narrative).	‘Moderate’The initial exercise training intensity is in the range of 40%–70% of a patient’s capacity. This is ideally based on ISWT or 6MWT calculated METs prior to commencing the core exercise training component.Each prescribed walking level is based on walk test distances or speeds with goals tailored to patient preferences.
	The allocated CBE level or WP pace or distance is validated by facilitators through Subjective checks using patient sensations (“make you breathe heavier, feel warmer and have a slightly faster heartbeat, but you should still be able to talk”) and Use of the REACH-HF manual tracker (0–10) effort scale where zero~no significant effort in carrying out the task to 10 representing excessive effort that is very difficult to maintain. Patients with facilitators are encouraged to understand and gain experience of the effort scale and try to avoid too many occasions where patients go above a rating scale of seven on the effort scale. If the effort required during a period of sustained exercise (eg, 3 or more minutes) is rated as eight or above, then the next exercise period (intensity level) should be adjusted down to a lower level.	

* Although the CBE has a defined warm-up period of 6 to 7 mins per session, all exercises in the main part of each CBE level are also steadily progressive, allowing the muscles, joints and physiological responses to adapt with each minute of the exercise.

### Usual care

Intervention and control patients will receive usual medical management as per clinical practice guidelines^3 5^ for treatment of participants with HFpEF. This includes the screening for both cardiovascular and non-cardiovascular comorbidities such as hypertension, diabetes mellitus, ischaemic heart disease and atrial fibrillation, which should be treated with safe and effective interventions that exist to improve symptoms, well-being and prognosis. Diuretics are recommended in those who are congested to alleviate symptoms. As part of usual care, all patients in the trial will be provided with the British Heart Foundation ‘Living with heart failure’ booklet.^16^ At the 4-month and 12-month follow-up, we will record any cotherapies received as part of usual care.

### Outcome measures

All primary and secondary outcomes will be collected at baseline (prerandomisation) and 4-month and 12-month postrandomisation. At the time of follow-up, patients will be asked if they have had any adverse events. The principal investigators (PIs) will be required to report serious adverse events within 24 hours of becoming aware of the event to the pharmacovigilance office. Any serious adverse events occurring during the trial will be recorded and reported to the Ethics Committee and the Data Monitoring Committee.

#### Primary outcome

Patient disease-specific HRQoL data will be collected at 12 months postrandomisation through the MLwHF Questionnaire. This validated questionnaire consists of 21 items to assess the impact of living with HF on the key physical, emotional, social and mental dimensions of quality of life.^17^ It provides scores for two dimensions, physical and emotional, and a total score.

#### Secondary outcomes

Patients:

- Exercise capacity (incremental shuttle walk test).^18^
- Physical activity levels (accelerometry over a 9-day period, measured using the GENEActiv Original accelerometer).^19^
- Psychological well-being measured using the Hospital Anxiety and Depression Scale questionnaire^20^
- Generic health-related quality of life using EuroQol EQ-5D-5L questionnaire^21^
- Generic health-related quality of life Short-Form-12 (SF-12)^22^
- Kansas City Cardiomyopathy Questionnaire^23^
- Frailty using the Clinical Frailty Scale^24^
- Self-care of HF Index questionnaire^25^
- Self-efficacy for key behaviours questionnaire.^11^
- Biomarker of cardiac wall stress NT-proBNP level.
- Clinical events assessed by deaths and hospital admissions (with HF-relatedness determined by an independent adjudication panel).

Caregivers:

- Caregiver burden for HF Questionnaire.^26^
- Caregiver contribution to Self-care of HF Index questionnaire.^27^
- Family caregiver Quality of Life Scale questionnaire.^28^
- Generic health-related quality of life using EQ-5D-5L.^21^
- Psychological well-being using the HADS questionnaire.^24^

**Figure 3:**
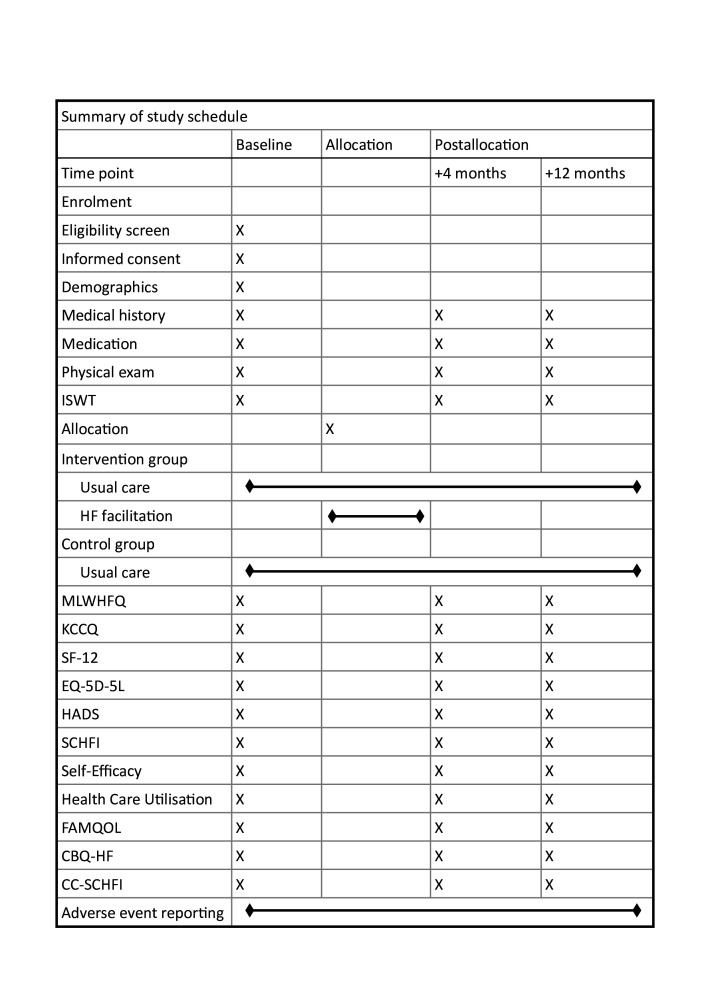
Summary of the study schedule. ISWT, Incremental Shuttle Walk Test; MLWHFQ, Minnesota Living with Health Failure Questionnaire; KCCQ, Kansas City Cardiomyopathy Questionnaire; SF-12, Short Form 12; HADS, Hospital Anxiety and Depression Scale; SCHFI, Self-Care of Heart Failure Index; FAMQOL, Family Caregiver Quality of Life Scale; CBQ-HF, Caregiver Burden Questionnaire for Heart Failure; CC-SCHFI, Caregiver Contribution to Self-Care of Heart Failure Index.

Summary of the study schedule is detailed in figure 3.

**Sample size**

At the design stage, the trial sample size was calculated in accordance with the DELTA2 guidance.^29^ A total of 520 (260 per group) participants with HFpEF is required for 90% power at 5% significance to detect a mean difference on the MLwHF Questionnaire of 5 points,^17^ assuming a SD of 20 points,^13^ a within patient correlation of 0.59 between baseline and 6 month follow-up, and an attrition rate of 15%. A 5-point difference in MLwHFQ score represents a minimum clinically important difference. Data from the REACH-HFpEF pilot trial^13^ indicate that the correlation between baseline and 6 months will be at least 0.59 (estimated correlation 0.73, 95% CI: 0.59 to 0.83).

Prior to the final analysis, in January 2025, the trial sample size was reassessed. A recent publication^30^ examining the responsiveness and minimal clinically important difference (MCID) of the MLwHF questionnaire suggested that a 16.6-point improvement represents a favourable outcome for patients. Based on a blinded access to trial data, specifically the overall distribution of changes in MLwHF scores, it was calculated that a mean between-group difference of 6.7 points in score at 12 months would equate to 50% more patients achieving a favourable outcome. Taking this as a MCID between groups, combined with the current baseline-adjusted residual SD in 12-month MLwHF scores of 17.8 points, and the current 12-month retention rate of 81%, the required sample size for 90% power at 5% significance was calculated to be 372. The rationale and basis of this updated sample size calculation were reviewed and approved by the Trial Steering Committee (TSC), Data Monitoring Committee (DMC) and trial patient & public involvement (PPI) group.

### Trial data collection

All required study data will be captured in a set of purpose-built eCRFs. Access to the eCRFs will be restricted, via a trial-specific web portal, and only authorised personnel will be able to enter data. The site principal investigator or their designee(s) will be responsible for all entries into the eCRF and will confirm that the data are accurate, complete and verifiable. Data will be stored in a Microsoft SQL Server database at the University of Glasgow Clinical Trials Unit, which has an ISO 9001 quality management system and ISO 27001 for information security.

Participants will be able to complete their questionnaires on a paper CRF (that will then be entered into the eCRF by the local research team) or to complete them electronically. Where completed electronically, data will be entered directly into a participant-facing version of the eCRF. As the eCRF will be adapted for self-completion, consent will be sought to use the participant contact details provided for recontact to verify responses as needed. Participants who consent to long-term follow-up of their outcomes using routine data, NHS/Community Health Index numbers will be collected to facilitate the potential collection of data in the future.

Regular data management/cleaning will be undertaken to assess data quality. Quality assurance checks will be performed to monitor the level of missing data and the timeliness of data entry and check for inconsistent data.

### Process evaluation

The process evaluation will assess the following research questions:

1. Was the intervention delivered as intended?
2. What adaptations were made/required in the intervention and do these impact outcomes?
3. Was the intervention used as intended?
4. What mechanisms explain any observed impact on patients’ HRQoL and other patient and caregiver outcomes?
5. What are the perspectives of patients, caregivers and service providers on the experience of being involved in REACH-HF?
6. What factors are associated with variation in intervention effectiveness among intervention recipients?
7. What adaptations were made within the service and did these impact fidelity and outcomes?

The process evaluation will use mixed methods at multiple case levels (patient, facilitator and centre) to test the programme theory in the population with HFpEF, identifying which components and configurations are best suited to meet their needs.^31 32^ The process evaluation will identify refinements of the programme theory, to optimise implementation and ensure that the essential ingredients of future interventions are better identified, interrogated and tested.^33^ As the analysis progresses, the implementation strategy will be revisited, focusing on potential outcomes such as Non-adoption, Abandonment, Scale-up, Spread, Sustainability Framework.^34^ This will maximise the clinical application of our research findings and enhance the capacity of staff working with participants with HFpEF to implement the intervention.

The participants in this process evaluation will comprise a subsample of patients, caregivers and REACH-HF facilitators taking part in the REACH-HFpEF trial. To answer the research questions, this mixed-method process evaluation will use trial primary and secondary outcomes and collect additional qualitative data (eg, intervention session recordings and interviews). The process evaluation will use multimodal longitudinal data.^35 36^

#### Process evaluation 1: participant and caregiver experience

15–20 patients (and 10 caregivers of these same patients) will be purposively selected and invited to take part in semistructured interviews. Patients will be chosen to represent, for example, diversity in terms of site/facilitator, sex, ethnicity, presence of a caregiver and baseline MLwHF Questionnaire total score.

The research team will interview each of these patients/caregivers at 4 months after the baseline visit (ie, immediately after intervention delivery is complete) and 12 months after the baseline visit. This will allow capture of patient and caregiver narratives over time, in relation to both intervention receipt and the longer-term impact/maintenance of self-care following the intervention. We will audio or video record these interviews, which may be conducted in person (if possible) or remotely (if not). Recording will use encrypted recording methods (either via password-protected online meeting software or an encrypted voice recorder). Written consent will be obtained prior to face-to-face interviews.

Topic guides for the interviews have been codeveloped with the patient and public involvement (PPI) advisory group. Interviews are designed to last between 30 min and 60 min. The researcher will endeavour to interview the patients without the caregiver present, where possible, and be mindful of the patient’s symptoms, such as fatigue or breathlessness, which may make an interview burdensome for the participant. The two interviews (and potentially selected segments of the intervention session recordings which represent good practice) will be transcribed verbatim. Thus, for each patient, their qualitative dataset is likely to comprise: two face-to-face meetings with their facilitator, five telephone meetings with their facilitator and two interviews with the process evaluation team.

#### Process evaluation 2: REACH-HF facilitator’s experience

In addition, 15 REACH-HF facilitators will be invited to take part in the process evaluation.

The process evaluation team will send an email to the participating facilitators with a brief questionnaire about their clinical background. This short questionnaire will either be completed in an electronic Word document and returned via email, or by following a link in the email to an electronic questionnaire (eg, using the electronic questionnaire platform Qualtrics). The process evaluation team will endeavour to sample REACH-HF facilitators to represent diversity in, for example, site, background training (eg, physiotherapy and nursing) and years of experience in delivery of cardiac rehabilitation (gathered using the clinical background questionnaire, see above). A topic guide will inform the interview, premised on the existing literature and gaps in current knowledge about intervention delivery. These interviews will be conducted either in person or remotely via telephone/web-call.

Verbatim interview transcripts will be organised and coded using MAXQDA. A framework analysis will be conducted, and sections of data relating to the aims of this research will be assigned a code that summarises the content either descriptively or interpretively. Codes with common features will be grouped together in themes, before finally being assigned to overarching themes. Where possible, data about self-reported behaviour from the interviews will be compared with observed behaviour evident in the intervention session recordings. A second qualitative researcher from the team will conduct independent analysis of a subset of the data. The researchers’ reflexive memo notes will enhance the integrity of the analysis.

The analysis will characterise patients’ and caregivers observed and self-reported responses to the intervention and link these responses to engagement with the intervention and perceived benefit, identifying interpersonal processes that shape the effectiveness or ineffectiveness of the intervention. At 4 months, patients’ and caregivers’ engagement with, response to and use of the REACH-HF manual will be characterised and differences between patients noted. At 12 months, overall use and benefit and maintenance of self-care behaviours and coping skills will be characterised and linked to individual differences in 4-month responses. Analysis will explore both patients’ and caregivers’ experiences of participation in the intervention and explicitly examine any potential impact of caregiver presence on patient adherence to the REACH-HF intervention.

#### Process evaluation 3: fidelity of intervention delivery

Facilitator-patient interactions (face-to-face and phone) for up to 60 patients will be audio-recorded (approximately 5–6 interactions taking 4–5 hours per patient). Recordings will be assessed using a previously developed and tested fidelity assessment checklist.^8^ The 12-item checklist uses a 0–5 rating scale based on the Dreyfus scale for assessing clinical competence.^37^ It focuses on assessing the quality of delivery of key delivery processes, such as the use of a patient-centred communication style, making a plan of action and encouraging self-monitoring of progress (particularly with the exercise programme). Intervention delivery fidelity data will be presented descriptively (mean scores with SD or 95% CIs) and broken down by site and by facilitator (as well as the calculation of overall delivery fidelity scores) for each checklist item. This will clarify how well intervention components were delivered and may identify ways to optimise delivery for future implementation. It will also allow researchers to describe variability in fidelity of delivery across patients and facilitators.

In addition, segments of the recordings that represent clear examples of good practice associated with each component of delivery (each item on the checklist) will be identified by noting the start/end timestamps of the segment within the audio file. These segments will be transcribed and collated for informing future REACH-HF training. Any information that might be used to identify the patient or the facilitator within the transcript will be redacted.

We will report descriptive statistics to summarise the fidelity of intervention delivery for each checklist item and will (descriptively and anonymously) examine variations between sites. Synthesis of the analysis of the intervention delivery fidelity and the interview data will enable a qualitative evaluation of potential pathways and barriers to improvement, which will pay attention to discrepancies between expected and observed outcomes, to understand how context influences outcomes and to provide insights to aid future implementation.

#### Process evaluation 4: facilitator checklist and log

REACH-HF facilitators will be asked to complete a brief self-rated fidelity checklist after each session they deliver. This comprises questions about the same 12 delivery fidelity components described above and allows the facilitators to rate the occurrences of each feature (absence, minimal, some, sufficient, good, very good and excellent). An independent observer rating is resource-intensive, while self-rated assessment may provide a pragmatic, real-world alternative to monitor delivery quality. The validity of the self-rating method will be checked by examining the correlation with observer-rated intervention delivery fidelity. We will also explore in the qualitative interviews whether use of the checklist facilitates/encourages reflexive practice and, in doing so, the quality of implementation.

Additionally, facilitators will be asked to complete a facilitator contact log for each participant. This log is a one-page pro forma designed to capture time, expenditure and any other resources required for the implementation of REACH-HF, as well as any adaptations made to the intervention for individual patients. It will capture data for both assessment of the fidelity of REACH-HF delivery and economic analyses.

A detailed process evaluation analysis plan will be drafted prior to study data lock and agreed with the Trial Management Group (TMG) and TSC.

### Economic evaluation

Economic analysis will be performed to establish the cost-effectiveness of REACH-HF plus usual care compared with usual care alone. Following on from the results of the economic evaluation pilot study,^13^ a within-trial cost-utility analysis will be conducted. Pilot study findings revealed differential resource distributions across primary, secondary and social care as well as impacts on informal carer time and costs. Bespoke data capture instruments have been developed to ensure capture of all relevant resource use from both an NHS/Personal Social Services (PSS) perspective, as well as a broader societal perspective. There is evidence of insensitivity of the EQ-5D-5L in patients with mild HF.^3638^,^41^ A recent study comparing the EQ-5D-5L and short-form six-dimension (SF-6D) in elderly participants with HF recommends use of SF-6D in those with milder disease and economic outcomes.^39^ Therefore, we propose to use both the SF-6D (from SF-12) and the EQ-5D-5L. As recommended by NICE economic evaluation guidance, the base-case perspectives will be that of the UK NHS and PSS.^42^ Further, a broader societal perspective, accounting for resource use, productivity (employment) and personal cost impacts faced by patients and their carers will be considered in sensitivity analyses, along with a scenario analysis incorporating HRQoL values obtained from mapping MLwHF Questionnaire scores to EQ-5D utilities, using a validated mapping algorithm.^43 44^ The base case economic evaluation will estimate the incremental cost per QALY associated with the REACH-HF intervention, compared with usual care alone, and will be reported in line with updated reporting guidelines for economic evaluations.^45^ The wider societal perspective will incorporate resource use, productivity (employment) and personal costs. Missing resource use and outcome data will be handled using multiple imputation.^46^ If within-trial results reveal between-group differences in HRQoL, a decision analytic model will be developed to estimate the cost-effectiveness results over a lifetime horizon.

A detailed health economic analysis plan will be drafted prior to study data lock and agreed with the TMG and TSC.

### Statistical analysis

Participation from screening to completion of the final follow-up assessment will be reported. Baseline patient characteristics and outcome scores will be summarised descriptively.

The primary statistical analysis for both primary and secondary outcomes will take an intention to treat approach (according to randomised allocation) based on complete data. For continuous outcome measures, mixed-effects regression will be used with a random effect of recruiting site and adjusting for baseline outcome score and minimisation variables. Additional clustering of outcomes due to therapist effects will be accounted for in sensitivity analyses.

A number of secondary analyses will be undertaken. Patterns and reasons for missing outcome data will be assessed, and sensitivity analyses will use appropriate imputation models to assess the impact of missing data. Potential subgroup treatment effects will be explored by adding treatment-by-subgroup interaction terms to analysis models. Potential subgroups assessed will include sex, study site and participant baseline NT-proBNP levels, ejection fraction and important markers of inequity, such as age, socioeconomic status and having a carer. Since the trial is powered to detect overall differences between the groups rather than interactions of this kind, these subgroup analyses will be regarded as exploratory. Before the start of recruitment, the TMG (with TSC approval) will be asked to define the minimum adherence to the REACH-HF intervention required to indicate compliance. Complier average causal effects analyses will be used to estimate the causal intervention effect in relation to each outcome.

Adherence will be defined using criteria adapted for the delivery processes proposed for the current study. These criteria will be developed with the TMG, building on the criteria used in the prior multicentre REACH-HF trial in people with HFrEF.^11^ Associations between physiological, cognitive and demographic factors and intervention adherence will be explored.

Estimated between-group differences will be presented using both absolute and relative measures, with associated 95% CIs, where appropriate. No correction of p values for multiplicity of testing will be undertaken. However, the analysis for the primary outcome will be performed before all other analyses, and the p values of all subsequent analyses will be interpreted in the context of multiple testing. No interim analyses are planned. Safety/adverse event outcomes will be reported descriptively by group.

A detailed statistical analysis plan will be drafted prior to study data lock and agreed with the TMG and TSC.

### Substudies

Three prespecified substudies are being undertaken alongside the main REACH-HF trial.

- Study within a trial (SWAT): the objective of the SWAT is to determine if an evidence-based enhanced participant information sheet impacts on recruitment and retention of caregivers to a multicentre host trial. Embedded in the main trial, the SWAT will be a cluster RCT design with allocation of the trial sites to either the enhanced host trial caregiver PIS (SWAT intervention group) or the standard host trial caregiver PIS (SWAT control group). The SWAT is led by University College Dublin and is registered with the ISRCTN trial registry (ISRCTN15757498) and the MRC SWAT Repository (https://www.qub.ac.uk/sites/TheNorthernIrelandNetworkforTrialsMethodologyResearch/FileStore/Filetoupload,1218962,en.pdf). The SWAT protocol will be submitted for publication separately.
- Optimisation of exercise fidelity in home-based cardiac rehabilitation study. This substudy aims to apply novel indicators of exercise fidelity (ie, quality of exercise in relation to the exercise prescribed) in the participants with HFpEF participating in the main trial. By identifying measurable indicators of exercise fidelity and associating them with patient outcomes, the substudy intends to identify ways to assess and tailor future home-based exercise interventions. Assessing the quality of the patients' exercises might also give them useful feedback about their progress and how they can get more benefit from the exercise component in future implementations of the REACH-HFpEF (or other home-based exercise interventions). This substudy is led by the University of Birmingham.

This substudy will seek a sample of up to 80 intervention group patient participants with a tracker watch and mobile phone and a brief questionnaire. These will be used to: (a) measure resting heart rate pre and post intervention (b) monitor heart rate during all their REACH-HFpEF exercise sessions and (c) video-record 1–2 exercise sessions to check for safety and accuracy of Quantitative

Mediation Analysis: the proposed statistical mediation sub-study will form an extension of the main trial process evaluation and aims to assess the association of the change of secondary outcomes as potential mediators of the REACH-HFpEF intervention primary outcome measure (MLwHF questionnaire). This substudy is led by the University of Exeter.

### Data monitoring and quality assurance

Trial-specific work instructions will be developed in accordance with University of Glasgow Clinical Trial Unit procedures. Regular data management and cleaning will be undertaken to assess data quality. Quality assurance checks will be undertaken to monitor the level of missing data and the timeliness of data entry and check for illogical or inconsistent data. The research team will monitor data collection procedures, ensuring that study data entry procedures are followed. The sponsor has categorised this trial as low risk and will therefore not be routinely monitored. The trial may be subject to audit by the sponsor.

### Trial management and independent committees

The Trial Operations Group (TOG) team members directly involved with the day-to-day running of the trial (co-chief investigators (CC/RST) and trial managers (EB/COH/AP/ET) and trial administrator) will meet on a 2-week basis to monitor and discuss the day-to-day management and all aspects of progress of the study. The TOG will have regular contact with trial sites by email and webinar meetings. The TMG, including the health economics, statistics, process evaluation teams, co-applicants and PPI representation, will meet on a termly basis to review the status of the study and trial progress.

The REACH-HFpEF TSC consists of independent members with clinical and trial methodological expertise and includes a patient and public involvement representative. The TSC will provide independent oversight of the conduct, timelines and funding of the trial with safety and ethics review by an independent DMC. The TSC and DMC will normally meet one to two times per year. Detailed descriptions of the remit and function of the committees are documented in specific charters held in the Trial Master File by Glasgow Clinical Trials Unit.

### Patient and public involvement

A PPI group will be established for this trial: 12 participants with lived experience of HFpEF and their partners/carers. These patients are usually managed and monitored in general practice.^12^ We will advertise on the NIHR People in Research website to recruit these patients and their partners/carers to the PPI group. An induction webinar will be held to introduce the group to the study and to negotiate characteristics of the PPI role throughout the study, including training and support needs.

Additionally, PPI representatives were members of the TMG and TSC.

## Ethics and dissemination

The study will be conducted in accordance with the ethical principles that have their origin in the Declaration of Helsinki and that are consistent with ICH GCP, and in accordance with the Research Governance Framework for Health and Social Care, Second edition (2005). The study and all relevant study documents have been reviewed and approved by the West of Scotland Research Ethics Service (reference number 21/WS/0085). The study sponsor is The NHS Greater Glasgow and Clyde. Written informed consent will be obtained from all study participants by the PI or designee prior to enrolment in the trial. All protocol modifications are being communicated to Research Ethics Committee (REC), funder, sponsor, TSC and DMC.

Study results will be published in open access publications in high impact peer-reviewed journals, including an end of trial NIHR monograph, and will be presented at national and international conferences. The study will be featured at the stakeholder dissemination workshop (with patients, clinicians, commissioners, academics and key groups such as British Heart Foundation, British Association for Cardiovascular Prevention and Rehabilitation and Pumping Marvellous). Direct feedback will be given to trial participants, and information will be digitally publicised on the REACH-HF website and relevant profiles on social media platforms.

### Trial status

The first participant with HFpEF was recruited in May 2022. The trial has opened 20 sites in England, Scotland and Wales (see appendix for listing) and as of 22nd May 2025 has recruited 382 participants with HFpEF and 94 caregivers.

## Supplementary material

10.1136/bmjopen-2024-094254 online supplemental file 1

10.1136/bmjopen-2024-094254 online supplemental file 2

## References

[1] Conrad N, Judge A, Tran J (2018). Temporal trends and patterns in heart failure incidence: a population-based study of 4 million individuals. Lancet.

[2] Braunwald E (2015). The war against heart failure: the Lancet lecture. Lancet.

[3] McDonagh TA, Metra M, Adamo M (2023). 2023 Focused Update of the 2021 ESC Guidelines for the diagnosis and treatment of acute and chronic heart failure. Eur Heart J.

[4] National Cardiac Audit Programme (2019). National heart failure audit: 2019 summary report (2017/18 data).

[5] National Institute for Health and Care Excellence (2018). Chronic heart failure in adults: diagnosis and management. NICE Guideline [NG106].

[6] Lam CSP, Donal E, Kraigher-Krainer E (2011). Epidemiology and clinical course of heart failure with preserved ejection fraction. Eur J Heart Fail.

[7] Anker SD, Butler J, Filippatos G (2021). Empagliflozin in heart failure with a preserved ejection fraction. N Engl J Med.

[8] Sebastian SA, Padda I, Johal G (2024). Supervised exercise training in heart failure with preserved ejection fraction: A systematic review and meta-analysis of randomized controlled trials. Curr Probl Cardiol.

[9] Greaves CJ, Wingham J, Deighan C (2016). Optimising self-care support for people with heart failure and their caregivers: development of the Rehabilitation Enablement in Chronic Heart Failure (REACH-HF) intervention using intervention mapping. *Pilot Feasibility Stud*.

[10] Taylor RS, Dalal HM, Zwisler AD (2023). Cardiac rehabilitation for heart failure: “Cinderella” or evidence-based pillar of care?. Eur Heart J.

[11] Dalal HM, Taylor RS, Jolly K (2019). The effects and costs of home-based rehabilitation for heart failure with reduced ejection fraction: The REACH-HF multicentre randomized controlled trial. Eur J Prev Cardiol.

[12] Taylor RS, Sadler S, Dalal HM (2019). The cost effectiveness of REACH-HF and home-based cardiac rehabilitation compared with the usual medical care for heart failure with reduced ejection fraction: A decision model-based analysis. Eur J Prev Cardiolog.

[13] Lang CC, Smith K, Wingham J (2018). A randomised controlled trial of a facilitated home-based rehabilitation intervention in patients with heart failure with preserved ejection fraction and their caregivers: the REACH-HFpEF Pilot Study. BMJ Open.

[14] Chan A-W, Tetzlaff JM, Altman DG (2013). SPIRIT 2013 statement: defining standard protocol items for clinical trials. Ann Intern Med.

[15] Hoffmann TC, Glasziou PP, Boutron I (2014). Better reporting of interventions: template for intervention description and replication (TIDieR) checklist and guide. BMJ.

[16] British Heart Foundation Living with heart failure.

[17] American Thoracic Society (2004). Minnesota living with heart failure questionnaire, New York.

[18] Pulz C, Diniz RV, Alves ANF (2008). Incremental shuttle and six-minute walking tests in the assessment of functional capacity in chronic heart failure. Can J Cardiol.

[19] van den Berg-Emons HJ, Bussmann JB, Balk AH (2000). Validity of ambulatory accelerometry to quantify physical activity in heart failure. Scand J Rehabil Med.

[20] Snaith RP (2003). The hospital anxiety and depression scale. Health Qual Life Outcomes.

[21] Herdman M, Gudex C, Lloyd A (2011). Development and preliminary testing of the new five-level version of EQ-5D (EQ-5D-5L). Qual Life Res.

[22] Gandek B, Ware JE, Aaronson NK (1998). Cross-validation of item selection and scoring for the SF-12 health survey in nine countries: results from the IQOLA Project. International quality of life assessment. J Clin Epidemiol.

[23] Green CP, Porter CB, Bresnahan DR (2000). Development and evaluation of the Kansas City cardiomyopathy questionnaire: a new health status measure for heart failure. J Am Coll Cardiol.

[24] Rockwood K, Song X, MacKnight C (2005). A global clinical measure of fitness and frailty in elderly people. Can Med Assoc J.

[25] Riegel B, Barbaranelli C, Carlson B (2019). Psychometric testing of the revised self-care of heart failure index. J Cardiovasc Nurs.

[26] Humphrey L, Kulich K, Deschaseaux C (2013). The Caregiver Burden Questionnaire for Heart Failure (CBQ-HF): face and content validity. Health Qual Life Outcomes.

[27] Vellone E, Barbaranelli C, Pucciarelli G (2020). Validity and reliability of the caregiver contribution to self-care of heart failure index version 2. J Cardiovasc Nurs.

[28] Nauser JA, Bakas T, Welch JL (2011). A new instrument to measure quality of life of heart failure family caregivers. J Cardiovasc Nurs.

[29] Cook JA, Julious SA, Sones W (2019). Practical help for specifying the target difference in sample size calculations for RCTs: the DELTA^2^ five-stage study, including a workshop. Health Technol Assess.

[30] Gonzalez-Saenz de Tejada M, Bilbao A, Ansola L (2019). Responsiveness and minimal clinically important difference of the Minnesota living with heart failure questionnaire. Health Qual Life Outcomes.

[31] Richards DA, Bazeley P, Borglin G (2019). Integrating quantitative and qualitative data and findings when undertaking randomised controlled trials. BMJ Open.

[32] Moore GF, Audrey S, Barker M (2015). Process evaluation of complex interventions: Medical Research Council guidance. BMJ.

[33] Craig P, Dieppe P, Macintyre S (2008). Developing and evaluating complex interventions: the new Medical Research Council guidance. BMJ.

[34] Greenhalgh T, Wherton J, Papoutsi C (2017). Beyond adoption: a new framework for theorizing and evaluating nonadoption, abandonment, and challenges to the scale-up, spread, and sustainability of health and care technologies. J Med Internet Res.

[35] Saldana J (2003). Longitudinal qualitative research.

[36] Guetterman TC, Fetters MD, Creswell JW (2015). Integrating quantitative and qualitative results in health science mixed methods research through joint displays. Ann Fam Med.

[37] Dreyfus HL, Burke J (1989). Competency based education and training.

[38] Kularatna S, Byrnes J, Chan YK (2017). Comparison of contemporaneous responses for EQ-5D-3L and Minnesota Living with Heart Failure; a case for disease specific multiattribute utility instrument in cardiovascular conditions. Int J Cardiol.

[39] Kularatna S, Byrnes J, Chan YK (2017). Comparison of the EQ-5D-3L and the SF-6D (SF-12) contemporaneous utility scores in patients with cardiovascular disease. Qual Life Res.

[40] Ambrosy AP, Cerbin LP, DeVore AD (2017). Aerobic exercise training and general health status in ambulatory heart failure patients with a reduced ejection fraction-Findings from the Heart Failure and A Controlled Trial Investigating Outcomes of Exercise Training (HF-ACTION)trial. Am Heart J.

[41] Flint K (2017). Cardiac rehabilitation in heart failure with reduced ejection fraction: A “take it or leave it” intervention. Am Heart J.

[42] NICE (2013). Guide to the Methods of Technology Appraisal 2013 London.

[43] Goldsmith KA, Dyer MT, Buxton MJ (2010). Mapping of the EQ-5D index from clinical outcome measures and demographic variables in patients with coronary heart disease. Health Qual Life Outcomes.

[44] Edlin R, Tsuchiya A, Brazier J (2002). Mapping the Minnesota Living with Heart Failure Questionnaire to the EQ-5D index.

[45] Husereau D, Drummond M, Augustovski F (2022). CHEERS 2022 ISPOR good research practices task force. consolidated health economic evaluation reporting standards 2022. Value Health.

[46] Glick H, Doshi J, Sonnad S (2007). Economic evaluation in clinical trials.

[47] Leventhal H, Diefenbach M, Leventhal EA (1992). Illness cognition: Using common sense to understand treatment adherence and affect cognition interactions. *Cogn Ther Res*.

[48] Deci EL, Ryan RM (1985). Intrinsic motivation and self-determination in human behavior.

[49] Wingham J, Harding G, Britten N (2014). Heart failure patients’ attitudes, beliefs, expectations and experiences of self-management strategies: a qualitative synthesis. Chronic Illn.

[50] Taylor SE (1983). Adjustment to threatening events: A theory of cognitive adaptation. American Psychologist.

[51] Carver CS, Scheier MF (1998). On the self-regulation of behavior.

[52] Beck AT (1989). Cognitive therapy and the emotional disorders.

[53] Sullivan MJ, Wood L, Terry J (2009). The Support, Education, and Research in Chronic Heart Failure Study (SEARCH): a mindfulness-based psychoeducational intervention improves depression and clinical symptoms in patients with chronic heart failure. Am Heart J.

[54] Hofmann SG, Sawyer AT, Witt AA (2010). The effect of mindfulness-based therapy on anxiety and depression: A meta-analytic review. J Consult Clin Psychol.

