# Supplementary material for: Clinical effectiveness and cost-effectiveness of the rehabilitation enablement in chronic heart failure facilitated self-care rehabilitation intervention for people with heart failure with preserved ejection fraction and their caregivers: rationale and protocol for a multicentre randomised controlled trial – REACH-HFpEF trial
Source: BMJ Open. 2025 May 27;15(5):e094254. doi: 10.1136/bmjopen-2024-094254 (PMC12121609; doi:10.1136/bmjopen-2024-094254)
Supplement: online supplemental file 1 [file bmjopen-15-5-s001.docx]

**Randomised controlled trial of a facilitated home-based rehabilitation intervention in patients with heart failure with preserved ejection fraction and their caregivers**

**(REACH-HFpEF)**

**Participant Consent Form**

| **CHIEF INVESTIGATOR**  Professor Rod Taylor and Professor Chim Lang  **PRINCIPAL INVESTIGATOR**  [INSERT LOCAL PI DETAILS HERE] |  |
| --- | --- |
|  | **Please initial box** |
| 1. I confirm that I have read and understood the information sheet (version x.x, dated xx/xx/xxxx) for the REACH-HFpEF study. I have had the opportunity to consider the information provided to me, ask questions and have had these answered satisfactorily. |  |
| 1. I understand that my participation in this study is voluntary and that I am free to stop taking part at any time without giving any reason and without my medical care or legal rights being affected. If I decide to stop participating in this study, I understand that any data already collected about me will be retained and used by the research team. |  |
| 1. I understand that relevant sections of my medical notes and data collected during the study will be looked at by individuals from the research team within the Universities of Glasgow, Exeter and Birmingham, NHS Tayside, from regulatory authorities, and from NHS Greater Glasgow and Clyde (the sponsor of the study), where it is relevant to my taking part in this research. I understand that this data will be held in a database at the University of Glasgow. |  |
| 1. I agree that my contact details (name, postal address, email address, and phone number) can be retained by the study team for use in relation to study procedures, and will be stored separately on a secure University of Glasgow server (secure online database). I understand that University of Exeter will be provided with my contact details to send me the GENEActiv accelerometer. |  |
| 1. I agree to the information I give being shared with other researchers for research and teaching, in line with a University of Glasgow data sharing agreement. I understand that anonymous data from my questionnaires will be deposited in a repository such as the UK Data Service, and that I will not be identified in any data shared. |  |
| 1. I agree to my blood samples being transferred to NHS Tayside, Dundee for analysis. |  |
| 1. I agree to my GP being informed about my participation |  |
| 1. I agree to take part in the REACH-HFpEF study. |  |

| **Optional Consents:** | | | | |  | |
| --- | --- | --- | --- | --- | --- | --- |
|  | | | | | **Yes/No** | |
| 1. I agree to be interviewed by a member of the research team about my experience in receiving the intervention and ways in which it can be improved. I understand that this recording will be held securely at the Universities of Birmingham and Exeter. I understand that members of the research team at the Universities of Birmingham and Exeter will have access to my contact details in order to facilitate this process. | | | | | Yes No | |
| 1. I agree that the study team may contact me again at a later date (after completion of the REACH-HFpEF study) to ask me to complete an additional follow-up questionnaire or provide related information. | | | | | Yes No | |
| 1. I understand that sessions with my REACH-HF facilitator may be audio recorded and listened to by the research team so that they can better understand delivery of the programme. I understand that these recordings will be held securely at the Universities of Birmingham and Exeter. | | | | | Yes No | |
| 1. I agree my anonymised audio recordings from the intervention delivery that are selected to represent good practice can be used for training and education purposes | | | | | Yes No | |
| 1. I agree to additional blood samples being taken and stored for use in future, ethically approved research. | | | | | Yes No | |
| 1. I agree to long term follow-up information by record linkage being collected on my future wellbeing and treatment from NHS and Government health records. I understand that this information will be stored confidentially and securely at the University of Glasgow for further analysis by approved researchers, up to a period of 10 years after the study has finished. I agree that personal details including my NHS/CHI number, date of birth and postcode can be used to facilitate this process. Researchers will only be allowed access to anonymised information. This may include information on prescriptions, hospitalisations and test results. | | | | | Yes No | |
| 1. I would like to receive a copy of the final study results | | | | | Yes No | |
| 1. I agree to be contacted about an exercise fidelity sub-study if I am offered the REACH-HF programme. I understand that my contact details (name, phone number and address) will be shared with the sub-study research team at the University of Birmingham to allow them to make contact with me. I understand that by agreeing to be contacted, I am under no obligation to take part in the sub-study. | | | | | Yes No | |
|  | |  |  |  |  | |
|  | |  |  |  |  | |
| Name of Patient (PRINT NAME) | |  | Date |  | Signature | |
|  | |  |  |  |  | |
|  | |  |  |  |  | |

*To be scanned together with confirmation of consent and a pdf file produced for the study records, with a copy being sent back to the patient, and a copy being inserted into the patient’s notes.*
