# Supplementary material for: Clinical effectiveness and cost-effectiveness of the rehabilitation enablement in chronic heart failure facilitated self-care rehabilitation intervention for people with heart failure with preserved ejection fraction and their caregivers: rationale and protocol for a multicentre randomised controlled trial – REACH-HFpEF trial
Source: BMJ Open. 2025 May 27;15(5):e094254. doi: 10.1136/bmjopen-2024-094254 (PMC12121609; doi:10.1136/bmjopen-2024-094254)
Supplement: online supplemental file 2 [file bmjopen-15-5-s002.docx]

**Randomised controlled trial of a facilitated home-based rehabilitation intervention in patients with heart failure with preserved ejection fraction and their caregivers**

**(REACH-HFpEF)**

**Caregiver Consent Form**

| **CHIEF INVESTIGATOR**  Professor Rod Taylor and Professor Chim Lang  **PRINCIPAL INVESTIGATOR**  [INSERT LOCAL PI DETAILS HERE] |  |
| --- | --- |
|  | **Please initial box** |
| 1. I confirm that I have read and understood this information sheet (version x.x dated xx/xx/xxxx) for the above REACH-HFpEF study. I have had the opportunity to consider the information provided to me, ask questions and have had these answered satisfactorily. |  |
| 1. I understand that my participation in this study is voluntary and that I am free to stop taking part at any time without giving any reason and without my medical care or legal rights being affected. If I decide to stop participating in this study, I understand that any data already collected about me will be retained and used by the research team. |  |
| 1. I understand that data collected during the study will be looked at by individuals from the research team within the Universities of Glasgow, Birmingham and Exeter, Trinity College Dublin, from regulatory authorities, and from NHS Greater Glasgow and Clyde (the sponsor of the study), where it is relevant to my taking part in this research. I understand that this data will be held in a database at the University of Glasgow. |  |
| 1. I agree that my contact details (name, postal address, email address, and phone number) can be retained by the study team for use in relation to study procedures, and will be stored separately on a secure University of Glasgow server (secure online database). |  |
| 1. I agree to my anonymised data being shared with other bona fide researchers, via a repository such as the UK Data Service, and in line with a University of Glasgow data sharing agreement. |  |
| 1. I agree to take part in the REACH-HFpEF study. |  |

| **Optional Consents:** | | | | |  | |
| --- | --- | --- | --- | --- | --- | --- |
|  | | | | | **Yes/No** | |
| 1. I agree to be interviewed by a member of the research team about my experience in receiving the intervention and ways in which it can be improved. I understand that this recording will be held securely at the Universities of Birmingham and Exeter. I understand that members of the research team at the Universities of Birmingham and Exeter will have access to my contact details in order to facilitate this process. | | | | | Yes No | |
| 1. I agree that the study team may contact me again at a later date (after completion of the REACH-HFpEF study) to ask me to complete an additional follow-up questionnaire or provide related information. | | | | | Yes No | |
| 1. I understand that sessions with my REACH-HF facilitator may be audio recorded and listened to by the research team so that they can better understand delivery of the programme. I understand that these recordings will be held securely at the Universities of Birmingham and Exeter. | | | | | Yes No | |
| 1. I agree my anonymised audio recordings from the intervention delivery that are selected to represent good practice can be used for training and education purposes. | | | | | Yes No | |
| 1. I would like to receive a copy of the final study results. | | | | | Yes No | |
|  | |  |  |  |  | |
|  | |  |  |  |  | |
| Name of Caregiver (PRINT NAME) | |  | Date |  | Signature | |
|  | |  |  |  |  | |
|  | |  |  |  |  | |

*To be scanned together with confirmation of consent and a pdf file produced for the study records, with a copy being sent back to the participant.*
