# Supplementary material for: Clinical effectiveness and cost-effectiveness of the rehabilitation enablement in chronic heart failure facilitated self-care rehabilitation intervention for people with heart failure with preserved ejection fraction and their caregivers: rationale and protocol for a multicentre randomised controlled trial – REACH-HFpEF trial
Source: BMJ Open. 2025 May 27;15(5):e094254. doi: 10.1136/bmjopen-2024-094254 (PMC12121609; doi:10.1136/bmjopen-2024-094254)
Supplement: Supplementary file 3 [file bmjopen-2024-094254.reviewer_comments.pdf]

PEER REVIEW HISTORY

BMJ Open publishes all reviews undertaken for accepted manuscripts. Reviewers are asked to complete a checklist review form and are provided with free text boxes to elaborate on their assessment. These free text comments are reproduced below.

ARTICLE DETAILS

Title (Provisional)

Clinical effectiveness and cost-effectiveness of the Rehabilitation Enablement in Chronic Heart Failure (REACH-HF) facilitated self-care rehabilitation intervention for people with heart failure with preserved ejection fraction and their caregivers: rationale and protocol for a multicentre randomised controlled trial

Authors

Taylor, Rod; Burrell, Emma; O'Hare, Claire; Thomson, Elizabeth Anne; Placzek, Anna; Bollen, Jessica Charlotte; Cleland , J; Cowie, Aynsley; Dalal, Hasnain M; Deaton, Christi; Doherty, Patrick Joseph; Dudman, Katie; Fraser, Heather; Frost, Julia; Greaves, Colin; Hartshorne-Evans, Nick; Hillsdon, Melvyn; Ibbotson, Tracy; Jarallah, Mohammad; Jolly, Kate; McConnachie, Alex; McIntosh, Emma; Smith, Valerie; Squire, Iain; Taylor, Louise; van Beurden, Samantha; Lang, Chim; REACH-HFpEF, Collaborators Group

---

This article was not externally reviewed at BMJ Open. Protocols that have been independently assessed prior to submission to BMJ Open are usually fast-tracked to publication on the grounds that further substantial changes will not be possible. This independent assessment will usually be external, independent review for both a substantial grant award from a non-commercial or government funder and ethics approval.
